# Optimizing an electromagnetic wave absorber for bi-anisotropic metasurfaces based on toroidal modes

**DOI:** 10.1038/s41598-024-59503-8

**Published:** 2024-04-16

**Authors:** Sina Aghdasinia, Hossein Allahverdizadeh, Ehsan Afkari, Behrouz Ahmadpour, Mohammad Bemani

**Affiliations:** Department of electric and Computer Engineering, University of Tabriz, Tabriz, 5166616471 Iran

**Keywords:** Wireless Power Transfer, Far-field WPT, Rectenna, Energy harvesting, Metasurface, Chiral particle, Physics, Electronics, photonics and device physics, Electrical and electronic engineering

## Abstract

The design and optimization of an electromagnetic wave absorber for far-field wireless power transmission (WPT) is the subject of this research study. The goal of the research is to effectively absorb energy from ambient RF electromagnetic waves without the usage of a ground plane by employing metasurfaces with chiral components.By integrating trioidal moments into the design theory, the objective is to create a metasurface that functions in two frequency bands and produces high-quality resonance. The study also explores the dual non-homogeneity property of structures, polarization tensor coefficients, and the electromagnetic response of non-homogeneous metasurfaces. Based on the relative orientation of induced fields and moments, it delves deeper into the two basic possibilities for dual non-homogeneous elements. The development of chiral metasurfaces and the notion of electromagnetic chirality and its implications for polarization properties are introduced.

## Introduction

Providing energy to operate daily life electronic devices has been a very important challenge for mankind, and many researchers in the related fields are focused on this topic. Wireless power transmission technology is a new method to renew the energy of electronic devices and also a solution for the faced challenges. This technology is based on power transmission through electromagnetic waves in the medium of free space and the surrounding space of daily life. The concept of wireless power transmission goes back to the findings of Nikola Tesla^1^ in the last century, and the emergence of microwave technology after him brought the first practical wireless power transmission system^2^ into academic communities, and made it a subject for research and development for researchers and developers of technology markets. Research on the classification of far-field power transfer has advanced rapidly with the emergence of artificial materials and structures known as metamaterials. Metamaterials have led to significant advancements in various technological fields and, particularly, in the manipulation of electromagnetic fields ^3^. A power transmission system in the category of far-field power transmission, known as an energy harvesting system in conventional research sources, has significant advantages compared to near-field coupling-based power transfer. One of these advantages is that the power transfer path is converted to common and long-distance routes. This research aims to enhance rectenna efficiency by utilizing a two-dimensional planar array, where its elements are recognized as unit cells of a metasurface. Instead of designing a high-gain directional antenna, the focus of this study is on improving efficiency through this planar array configuration^4^. In the most general case, if the induced surface electric current in metasurfaces is considered to be generated by both the electric and radiative magnetic fields, and also if the duality of this phenomenon, meaning that the magnetic induction is influenced by both the electric and radiative magnetic fields, is observed, such metasurfaces can be referred to as dual non-homogeneous medium. The electromagnetic response of a homogeneous metasurface is determined by the electric dipole moment or torroidal vector (P) and the induced magnetic dipole moment or toroidal vector (m,g) within the constituent unit cells forming the metasurface. In a comprehensive linear formulation, the relationships between the induced electric and magnetic dipole moments and the external fields at a given location can be written as follows:

1 $$\begin{aligned} \left[ \begin{array}{c} \textbf{p} \\ \textbf{m} \end{array}\right] =\left[ \begin{array}{ll} \overline{\overline{\alpha }}_{\textrm{ee} }&{} \overline{\overline{\alpha }}_{\textrm{em}} \\ \overline{\overline{\alpha }}_{\textrm{me}} &{} \overline{\overline{\alpha }}_{\textbf{mm}} \end{array}\right] \cdot \left[ \begin{array}{l} \textbf{E}_{\textrm{loc}} \\ \textbf{H}_{\textrm{loc}} \end{array}\right] \end{aligned}$$

which are electrical, electric-magnetic, magnetic-electric and magnetic polarization tensor coefficients, respectively. The electromagnetic response of non-magnetic natural materials is usually determined by the electrical polarization coefficient, while the other polarization coefficients can be neglected due to the extremely small dimensions of the constituent particles of the natural material. A general answer related to all the introduced polarization coefficients can be achieved when artificial materials and structures (metamaterials and metasurfaces) are designed in such a way that the dimensions of its components (unit cells) are comparable to the wavelength of the external fields in the position of the unit cell. Assuming that the components of a metasurface are reprocal, the following conditions are valid for the polarization tensor coefficients:

2 $$\begin{aligned} \overline{\overline{\alpha }}_{\textrm{ee} }={\overline{\overline{\alpha }}^T_{\textrm{ee}}},\quad \overline{\overline{\alpha }}_{\textrm{mm} }={\overline{\overline{\alpha }}^T_{\textrm{mm}}},\quad \overline{\overline{\alpha }}_{\textrm{em} }=-{\overline{\overline{\alpha }}^T_{\textrm{me}}} \end{aligned}$$

Which operator T performs the transpose operation on the tensor coefficients. It can be observed that the magnetic-electric polarization coefficient is completely determined by the electric-magnetic polarization coefficient, and it fully exhibits the reciprocal property. As mentioned, the non-zero and non-negligible values of these two coefficients indicate the dual non-homogeneity property of the structure.

According to Eq. (1), two basic scenarios can be defined for the dual non-homogeneous reciprocal elements, based on the relative orientation of the induced field and moment.

Recent advancements in the field of multifunctional metasurfaces, composed of compact unit cells, have opened up new possibilities in ultra-high-speed trigonometric operations^5^. A key development is the non-interleaved bidirectional Janus metasurface, which, with its broken mirror symmetry, encodes multiple functionalities in full-space scattering channels with different propagation directions and polarization, thereby exploiting four independent information channels^6^. The integration of deep learning has further broadened the scope, with the experimental demonstration of a compact optical trigonometric operator that responds to incident light source modes, generating accurate results in the output layer^5^. As the field evolves, these metasurfaces are anticipated to play a crucial role in a range of applications, from structured light conversion and optical imaging to multifunctional optical information processing^7,8^.

## Related works

### Surface-based electromagnetic wave absorber

#### A. The field vector and the induced moment vector are in the same direction

**Figure 1:**
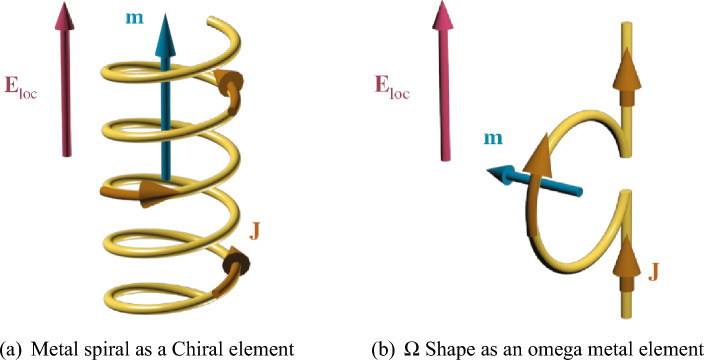
Two fundamental scenarios for nnon-homogeneous dual-responsive elements.

An induced current is generated under the influence of a vertical electric field, leading to the emergence of a magnetic moment aligned with the external electric field. This scenario occurs in spiral components where the current loop is perpendicular to the field radiation direction, as shown in Fig. 1a. In a general classification, chiral elements contribute to the emergence of the first scenario.

#### B.The field vector and the induced moment vector are orthogonal

The induced current by stimulation of a vertical electric field, forms a magnetic moment perpendicular to the external electric field. This scenario occurs in components and elements where the orientation of the formed current loop aligns with the direction of the field radiation. The second scenario is represented by omega elements, according to a general classification (Fig. 1b).

### Electromagnetic chirality

**Figure 2:**
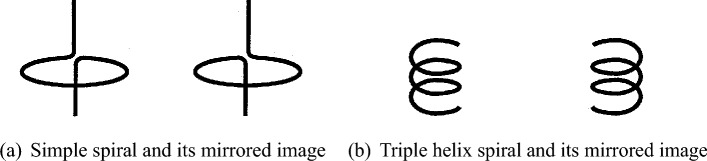
Examples of chiral elements.

Chirality is a geometric concept. We call a shape or object chiral when it cannot be superimposed onto its mirror image through translation and rotation; in other words, it lacks bilateral symmetry. A chiral element can be either right-handed or left-handed. Figure 2 shows a simple helix as a canonical example of a chiral element, which, if it exhibits right-handedness, its mirrored image exhibits left-handedness.

It is not surprising that the interaction between an electromagnetic wave and a collection of chiral objects and elements with distinct right-handed/left-handed properties leads to variations in the polarization properties of the electromagnetic wave. Such a process is known as electromagnetic chirality^9^.

### Metachirality

Chiral elements, which exhibit dual-handedness properties, can form structures called chiral metasurfaces. These metasurfaces consist of unit cells composed of chiral elements, such as simple helices. In a chiral metasurface, the chiral elements are arranged with a specific spacing distance, and their induced electric and magnetic dipole moments align in the same direction perpendicular to the metasurface. This configuration enables interaction with electromagnetic waves on the metasurface^10^.

### Toroidal dipole moments

Electric dipole moments arise from the separation of positive and negative electric charges, while magnetic dipole moments arise from closed loop currents. Mathematically, electric and magnetic dipole moments can be expanded into multipole moments of charge and current distributions, as described in^11^. The simplest radiation configuration that introduces the electric dipole moment is the separation of electric charges into positive and negative components. Inverting this configuration reverses the direction of the electric dipole moment, indicating that the electric dipole moment does not possess symmetry under this transformation. On the other hand, under such a coordinate inversion, the direction of the magnetic dipole moment does not change, as changes in the direction of the current compensate for the changes in spatial coordinates.

The concept of toroidal moment or toroidal multipole moments was not introduced in classical electrodynamics for the expansion of charge and current distributions until Yakov Zel’dovich, a physicist from the Soviet Union, challenged the conservation of parity in fundamental particles based on classical theory in 1957 and argued that the explanation for balance and parity requires the introduction of new concepts^12^. In 1967, Dubovik and colleagues associated these new concepts with classical electromagnetics by introducing “polar toroidal multipole moments,” represented by the toroidal multipole moment vector denoted as *t*^13^.

The term “toroidal” is used because it was first demonstrated that such a toroidal arises from the distribution of current on a structure in the form of a toroidal coil. Dubovik and colleagues showed that toroidal moments, such as electric and magnetic multipole moments, belong to a family of multipole moments. They attempted to express these moments using a classical expansion that existed for members of the old multipole family with the introduction of the polar toroidal moment, a new family of multipole moments potentially exists, called “Axial Toroidal Multipole Moments.” These moments exhibit vector properties in spatial coordinate transformation, and their dipole moment vector is defined as “*g*”^14^.

Although the axial toroidal moment may seem like a conceptual concept due to the absence of magnetic charge, its complete justification in electromagnetics was achieved in later years through non-linear optical effects in continuous media^15^. In later years, the toroidal moments were extensively analyzed and decomposed, and charge and current distributions leading to toroidal moments were introduced^16–18^. However, despite increasing theoretical understanding of this emerging concept, empirical and practical evidence of toroidal moments was scarce. The awareness of toroidal moments grew within the research community with the emergence of metamaterials in the new millennium. Metamaterials, composed of unit cells, exhibit strong electric permeability scattering for cells with a strong electric dipole response, and effective magnetic permeability scattering for cells with a strong magnetic dipole response. By induction, cells with strong toroidal moments should be identifiable^16^. In 2002, further analysis of the radiative properties of arbitrary charge and current distributions also considered the toroidal moment as a fixed member of the multipole moments. Its calculation was possible through the usual Cartesian coordinate expansion^19^.

**Figure 3:**
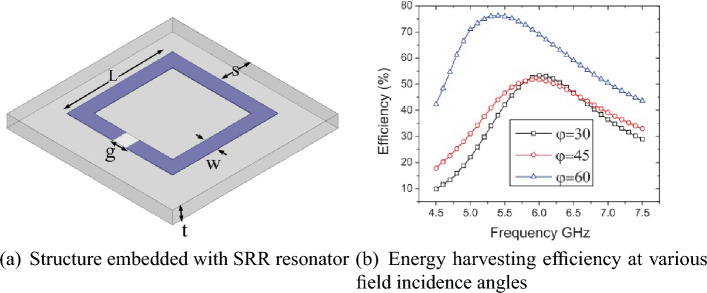
Reference^20^ results.

Figure 3 illustrates a unit cell of one of the first designed examples of surface-based absorbers for energy harvesting. Its efficiency is demonstrated at different angles of the incident radiation field within a specified frequency band shown in Figure 3-b.

**Figure 4:**
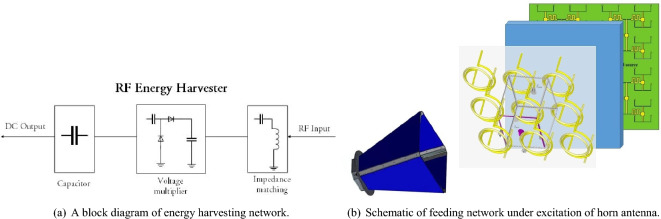
The output block diagram of energy harvesting network.

In terms of energy harvesting capability, the surfaces transfer the received power by each resonator to a rectifying circuit through a combining network. This concept has been discussed in several references^21–24^. The input impedance of each branch of the power combiner can be modeled with a resistance (along with a ground port). Therefore, in the design of surface energy harvesters, each unit cell is typically loaded with a grounded resistance^25,26^. In Fig. 4, you can see the simple schematic of an RF energy harvesting network.

The use of arrays of bow-tie cells has been another improvement in the field of energy harvesting, particularly in increasing the bandwidth. This has been discussed in reference^26^. Another fundamental advancement in this research area has been the significant improvement in achieving multi-band and multi-polarization capabilities in the designed surface arrays for energy harvesting^27–29^.

**Figure 5:**
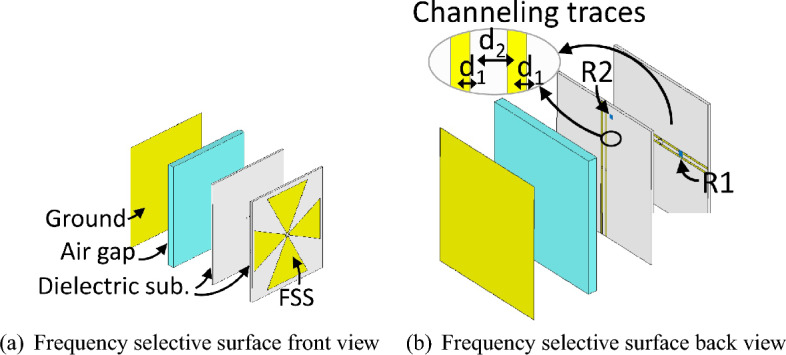
Embedded structure in Ref.^30^.

The approach and methodology presented in^30^ can be considered as an important and innovative effort in the field of multi-polarization energy harvesting in surface structures. Figure 5 illustrates the designed unit cell from the front view (Fig. 5a) and the back view (Fig. 5b) in this reference. It indicates the complexity of the design and the potential decrease in efficiency due to the complexity of the power transfer mechanism introduced by the absorbed power.

### Chiral elements for energy harvesting and optimization of these elements

Chiral surfaces have been introduced as an alternative to the reviewed surfaces that utilized resonators for designing the unit cell, requiring the presence of a ground plane and loading each cell through vias to create a rectifying network.

Polarizable electric-magnetic components of the same subwavelength wavelength serve as substitutes for using resonators in this category of surfaces for energy harvesting, and chiral elements are one type of these components for forming such a classification of surfaces.

In this section, the findings of^31^ regarding the analysis of a dual non-isotropic medium composed of chiral elements will be examined, along with its approach for extracting maximum power from electromagnetic fields. Ref.^31^ has formulated Eq. (3), which represents the dependency of the transverse component of the radiated field on the power at its own point of propagation

3 $$\begin{aligned} P=-\frac{\omega }{2} \text {Im} \{(\textbf{p} \cdot \textbf{E}_{\textrm{t}}^{*}+\textbf{m} \cdot \textbf{H}_{\textrm{t}}^{*}\} \end{aligned}$$

**Figure 6:**
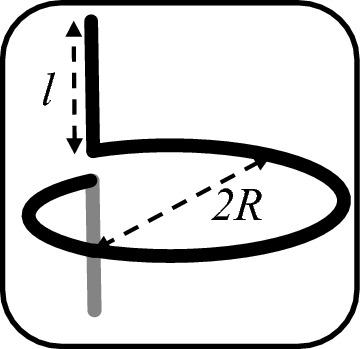
Each of the unit cells of Chiral Metasurface with radius *R* and length *l*.

For the classification of chiral elements as a category of dual non-isotropic response for interaction with electromagnetic waves, the Eq. (4) has utilized for the polarization coefficients of a simple helical chiral structure, as shown in Fig. 6^32^.

4 $$\begin{aligned} \alpha _{\textrm{ee}}&=-\textrm{j} \frac{l^2}{\omega Z_{\textrm{tot}}} \nonumber \\ \alpha _{\textrm{em}}&=\pm \textrm{j} \alpha _{\textrm{ee}} \eta _0 \frac{k_0 S}{l} \nonumber \\ \alpha _{\textrm{mm}}&=\alpha _{\textrm{ee}}\left( \eta _0 \frac{k_0 S}{l}\right) ^2 \end{aligned}$$

Assuming that *l* is the length of the element and $\omega$ is the angular frequency, $Z_{tot}$ represents the total impedance of a simple helical chiral structure as a chiral element, including the sum of the input impedance of the loop and the parts that correspond to a dual-polarized electric antenna. In classical sources such as ^33^ and modern antenna theory^34^, we should seek an equivalent for it. Assuming that this impedance value only has a real value at the resonance of the element, disregarding absorption losses and considering it dependent on scattering losses, the following real value can be presumed for it:

5 $$\begin{aligned} R_{\textrm{tot}}=2R_{Rad}=\frac{\eta _0}{3 \pi }\left( k_0^2 l^2+k_0^4 S^2\right) \end{aligned}$$

where $R_{Rad}$ is the radiation resistance of the element [38], and S is the area of the loop, given by $S=\pi R^2$, where *R* is the radius of the loop. $\eta _0$ and $k_0$ represent the impedance and wave number of free space, respectively.^31^ has obtained the optimal values for the conditions of a pure real wave with circular polarization as follows:

6 $$\begin{aligned} l_{\text{opt} }&=(2-\sqrt{3}) \frac{\lambda }{4}, \nonumber \\ R_{\text{opt} }&=\frac{\sqrt{3}-1}{\pi } \frac{\lambda }{4} \end{aligned}$$

The present research will investigate the power extracted from radiation under similar polarization conditions but with general propagation conditions, where the wave number includes an imaginary component, in follow.

**Figure 7:**
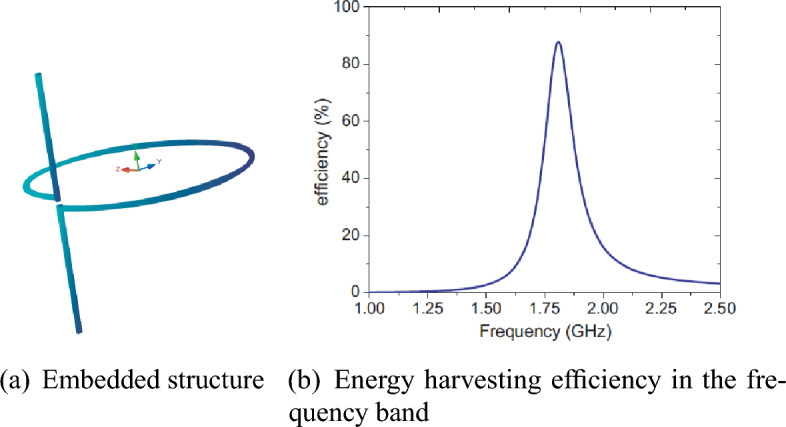
Reference^35^ results.

The source^35^ introduces a planar source with a unit cell consisting of a simple helix with $l=1.005\, \hbox {cm}$ and $R=0.874\hbox {cm}$, as shown in Fig. 7. This structure serves as a chiral element, creating a non-concentric dual-homogeneity environment.

These optimal values for the designed element correspond to the optimal dimensions derived in Eq. (6), which were obtained using an approach similar to that employed in Ref.^31^. However, in the present source, a closed-form equation for the optimal values of the chiral element is calculated.

This structure is specifically tailored for harvesting energy from electromagnetic waves with circular polarization within the surrounding environment. Unlike the reviewed examples in “Metachirality” , where surfaces composed of enhancers were studied as unit cells, this structure does not include surfaces coupled with the ground plane, and the absorbed energy is not transferred to the load through a via.

### Toroidal moment and classical multipole expansion

In the source^36^, a comprehensive analysis of the multipole expansion using vector potentials and electromagnetic fields is performed to establish a connection between the Toroidal and classical multipole expansion.

In summary, by comparing the electric field generated by an electric dipole E(p), which depends on the electric dipole moment, the coefficient 1/r or the radiation zone, the propagation direction vector n and the wave number k, as shown in Eq. (7)^11^, with the electric field calculated from the Toroidal multipole, E(t), as presented in Eq. (8) in the current source, a relationship between the Toroidal and classical multipole expansion is established.

7 $$\begin{aligned} \textbf{E}^{(p)}&=\frac{k^{2}}{4 \pi \epsilon _{0}}\left( \frac{1}{r}(\textbf{n} \times \textbf{p})\times \textbf{n} \right) \textrm{e}^{\textrm{j} k r} \end{aligned}$$

8 $$\begin{aligned} \textbf{E}^{(t)}&=\frac{1}{4 \pi \epsilon _{0}}\left( \frac{\textrm{i} k^{3}}{r}(\textbf{n} \times \textbf{t}) \times \textbf{n}+\left( \frac{\textrm{i} k}{r^{3}}+\frac{k^{2}}{r^{2}}\right) [3 \textbf{n}(\textbf{n} \cdot \textbf{t})-\textbf{t}]\right) \textrm{e}^{\textrm{i} k r} \end{aligned}$$

A general far-field electric field, denoted as $E^{(tot)}$, in the radiation zone is given by Eq. (9), which is consistent with the results in^42^.

9 $$\begin{aligned} \textbf{E}^{(t o t)} =\textbf{E}^{(p)}+\textbf{E}^{(t)}=\frac{k^{2}}{4 \pi \epsilon _{0}}\left( \frac{1}{r}\textbf{n} \times (jk\textbf{t}+\textbf{p})\times \textbf{n} \right) \textrm{e}^{\textrm{j} k r} \end{aligned}$$

Attention to the Eq. (9) clarifies the issue that choosing a charge and current distribution with magnetic dipole moment as $p = -jkt$ will result in the vanishing of the electromagnetic fields in the radiation zone. Although this disappearance of fields with such a distribution will also nullify the electromagnetic fields originating from the distribution in the equation $E^{(tot)}$, we did not address these fields. This conclusion confirms the existence of configurations known as “Anapoles” in the classical description, which refers to a distribution of charges and currents that neither radiates nor interacts with external electromagnetic fields^37^. It supports recent findings in the field of fundamental particle physics^38^.

The approach reviewed in this section will serve as a tool in incorporating toroidal moments in the calculations for designing electromagnetic wave absorbers based on metasurfaces in follow.

### Achieving high-quality factor through toroidal moment excitation

Resonance with a high-quality (*Q*) factor has garnered attention due to its positive attributes in electromagnetic sensing efficiency. It can also be beneficial in energy harvesting applications from electromagnetic waves^39^. Sub-radiant behavior of such resonances enhances power reception by trapping and maximizing electromagnetic energy in the near-field region of the power receiver.

One of the well-known methods to achieve high-*Q* resonance is through the interaction between bright and dark modes, as observed in resonance phenomena referred to as “Fano resonance”^40–42^. Increasing the *Q* factor can also be achieved by exciting two opposing toroidal moments within a metamolecule composing of metamaterials or periodic structures. The excitation of sub-radiant multipole moments of dark mode nature in periodic structures has been studied^43^.

However, investigating such excitations is highly challenging due to the difficulty in observing and characterizing the geometry-dependent toroidal moments and excited modes. Consequently, previous studies in this field have been largely limited to the regime of optical waves, where studying the interaction of waves with very short wavelengths with materials and microscale structures has been practical^44^.

Although the microwave regime has also been studied for the development of the concept of nonradiative mode excitation, thanks to the extensive possibilities for modifying and manipulating structures^45^, breaking the symmetry inside unit cells of metasurfaces, which have no continuous unit structure and allow for the creation of opposing and coherent toroidal dipole moments within them, has been explored^46^.

## Method and results

### Design of optimized single-cell

In the most general case, an electromagnetic wave absorber based on surfaces exhibits linear relationships between induced moments and incident radiation fields for each individual unit cell on the surface, which can be expressed in the following Eq. (1). Here, $E^{loc}$ and $H^{loc}$ represent the localized electromagnetic fields of the assumed plane wave radiation on the surfaces. To ensure a homogeneous response for each wave polarization and surface orientation, the surface is assumed to have uniaxial symmetry. Such symmetry allows the division of the tensorial polarizability values in Eq. (1) into coefficients of the transverse unit tensor $\overline{\overline{I_t}}$ and the perpendicular unit tensor $\overline{\overline{J_t}}$. These coefficients are referred to as co-directional and cross-directional polarizability coefficients, respectively. Please note that the response of the assumed elements or unit cells to the incident radiation fields is determined in the plane perpendicular to the radiation direction^47^.

10 $$\begin{aligned} \begin{array}{ll} \overline{\overline{{\alpha }}}_{e e}={\alpha }_{e e}^{\textrm{co}} \overline{\overline{I}}_{t}+{\alpha }_{e e}^{\textrm{cr}} \overline{\overline{J}}_{t}, &{} \overline{\overline{{\alpha }}}_{m m}={\alpha }_{m m}^{\textrm{co}} \overline{\overline{I}}_{t}+{\alpha }_{m m}^{\textrm{cr}} \overline{\overline{J}}_{t}, \\ \overline{\overline{{\alpha }}}_{e m}={\alpha }_{e m}^{\textrm{co}} \overline{\overline{I}}_{t}+{\alpha }_{e m}^{\textrm{cr}} \overline{\overline{J}}_{t}, &{} \overline{\overline{{\alpha }}}_{m e}={\alpha }_{m e}^{\textrm{co}} \overline{\overline{I}}_{t}+{\alpha }_{m e}^{\textrm{cr}} \overline{\overline{J}}_{t}, \end{array} \end{aligned}$$

The determination of these coefficients depends on the element type with which the interaction of radiated fields is examined. Chiral elements, which will be used to extract maximum power from electromagnetic radiation fields in the optimal design of unit cells, do not have a reciprocal component in their polarizability due to the property of reciprocity in the coupling of electric and magnetic moments. Because the assumed element’s response to the radiated fields occurs in the $x-y$ plane (with the *z* axis chosen as the symmetry axis), the electric and magnetic fields of the incident plane wave separate into transverse and normal components upon encountering the surfaces of the unit cell in time.

11 $$\begin{aligned} \textbf{H}^{\textrm{loc}}= & {} \textbf{H}_{\textrm{t}}+H_{z} \hat{\textbf{a}}_z \nonumber \\ \textbf{E}^{\textrm{loc}}= & {} \textbf{E}_{\textrm{t}}+E_{z} \hat{\textbf{a}}_z \end{aligned}$$

Plane wave radiation is considered to be parallel to the symmetry axis and perpendicular to the surfaces in all stages of optimal unit cell design. Therefore, the interaction of these fields with the polarizable element assumes that the fields **D** and **B** are separable correspondingly into transverse and normal components. The transverse components of the electric and magnetic fields are assumed to be as follows:

12 $$\begin{aligned} \textbf{H}_{\textrm{t}}= & {} H_{x} \hat{\textbf{a}}_x +H_{y} \hat{\textbf{a}}_y \nonumber \\ \textbf{E}_{\textrm{t}}= & {} -\overline{\overline{Z}} \cdot \hat{\textbf{a}}_z \times \textbf{H}_{\textrm{t}} \end{aligned}$$

If such a separation is done for the wave vector of this plane wave, such that we have $k={{k}_{t}}+\beta {{\hat{a}}_{z}}$, then the wave impedance $\overline{\overline{Z}}$ can be obtained ^47^.

13 $$\begin{aligned} \overline{\overline{Z}}=Z_{\textrm{TM}}\frac{\textbf{k}_{\textrm{t}} \textbf{k}_{\textrm{t}}}{k_{\textrm{t}}^{2}}+Z_{\textrm{TE}} \frac{\textbf{k}_{\textrm{t}} \times \hat{\textbf{a}}_z \textbf{k}_{\textrm{t}} \times \hat{\textbf{a}}_z}{k_{\textrm{t}}^{2}} \end{aligned}$$

The characteristic impedances $Z_{TM}$ and $Z_{TE}$ in relation to the wave impedance are equal to:

14 $$\begin{aligned} Z_{\textrm{TM}}=\frac{\beta }{\omega \epsilon _{0}}, Z_{\textrm{TE}}=\frac{\omega \mu _{0}}{\beta }. \end{aligned}$$

The linear relationship between electric and magnetic moments and transverse fields can be simplified as follows:

15 $$\begin{aligned} \textbf{p}= & {} \alpha _{\textrm{ee}}^{\textrm{co}} \textbf{E}_{\textrm{t}}+\alpha _{\textrm{ee}}^{\textrm{cr}} \hat{\textbf{a}}_z \times \textbf{E}_{\textrm{t}}+\alpha _{\textrm{em}}^{\textrm{co}} \textbf{H}_{\textrm{t}}+\alpha _{\textrm{em}}^{\textrm{cr}} \hat{\textbf{a}}_z \times \textbf{H}_{\textrm{t}} \nonumber \\ \textbf{m}= & {} \alpha _{\textrm{me}}^{\textrm{co}} \textbf{E}_{\textrm{t}}+\alpha _{\textrm{me}}^{\textrm{cr}} \hat{\textbf{a}}_z \times \textbf{E}_{\textrm{t}}+\alpha _{\textrm{mm}}^{\textrm{co}} \textbf{H}_{\textrm{t}}+\alpha _{\textrm{mm}}^{\textrm{cr}} \hat{\textbf{a}}_z \times \textbf{H}_{\textrm{t}} \end{aligned}$$

To optimize the design, we consider a unit cell consisting of two chiral elements with a distance of *d* between them. For each separate electric moment $p_1$, $p_2$, and magnetic moment $m_1$, $m_2$, it is assumed that the polarizabilities are unaffected by the mutual component interactions.

16 $$\begin{aligned} \begin{array}{ll} \textbf{p}_1=\alpha _{\mathrm {ee.1}}^{\textrm{co}} \textbf{E}_{\textrm{t}}+\alpha _{\mathrm {em.1}}^{\textrm{co}} \textbf{H}_{\textrm{t}}, &{} \textbf{p}_2=\alpha _{\mathrm {ee.2}}^{\textrm{co}} \textbf{E}_{\textrm{t}}+\alpha _{\mathrm {em.2}}^{\textrm{co}} \textbf{H}_{\textrm{t}}, \\ \textbf{m}_1=\alpha _{\mathrm {me.1}}^{\textrm{co}} \textbf{E}_{\textrm{t}}+\alpha _{\mathrm {mm.1}}^{\textrm{co}} \textbf{H}_{\textrm{t}}, &{} \textbf{m}_2=\alpha _{\mathrm {me.2}}^{\textrm{co}} \textbf{E}_{\textrm{t}}+\alpha _{\mathrm {mm.2}}^{\textrm{co}} \textbf{H}_{\textrm{t}}, \end{array} \end{aligned}$$

**Figure 8:**
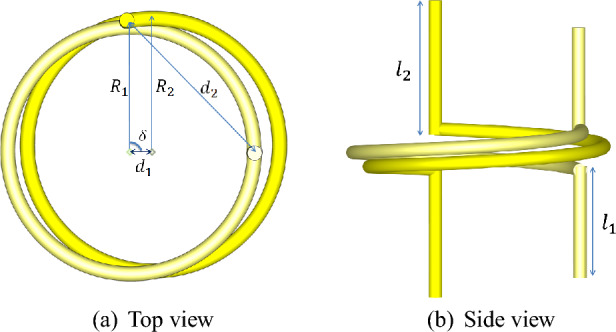
Proposed unit cell.

The magnetic moment pair $m_1$, $m_2$ generates a toroidal moment at a distance $d_1$ between them, which can be obtained using the following general equation^48^.

17 $$\begin{aligned} \textbf{t} =\frac{1}{2}\sum _{\alpha } {{\textbf{r}_\alpha } \times {\textbf{m}_\alpha }} \end{aligned}$$

And simplifying our problem, it can be expressed as follows:

18 $$\begin{aligned} \textbf{t} =\frac{1}{2} \Bigg [{\textbf{r}_{\textrm{m1}} } \times {\textbf{m}_1 }+{\textbf{r}_{\textrm{m2}} } \times {\textbf{m}_2 }\Bigg ]=\frac{1}{2} \Bigg [\frac{d_1}{2} \hat{\textbf{a}}_z\times \textbf{m}_1-\frac{d_1}{2} \hat{\textbf{a}}_z\times \textbf{m}_2\Bigg ] \end{aligned}$$

The vectors $r_{m1}$ and $r_{m2}$ represent the positions from the location of the first and second magnetic moments to the location where the toroidal moment *t* is formed. Additionally, the toroidal moment *g* created by the electric moment pair at a distance $d_2$ can be determined as the dual of Eq. (19) using Eq. (17).

19 $$\begin{aligned} \textbf{g} =\frac{1}{2}\Bigg [{\textbf{r}_{\textrm{p1}} } \times {\textbf{p}_1}+{\textbf{r}_{\textrm{p2}} } \times {\textbf{p}_2}\Bigg ]=\frac{1}{2} \Bigg [\frac{d_2}{2} \hat{\textbf{a}}_z\times \textbf{p}_1-\frac{d_2}{2} \hat{\textbf{a}}_z\times \textbf{p}_2\Bigg ] \end{aligned}$$

The vectors $r_{p1}$ and $r_{p2}$ represent the positions from the location of the first and second electric moments to the location where the toroidal moment is formed. By substituting the equations into Eq. (16) the expressions for the new moments, For the toroidal moment *t* we have:

20 $$\begin{aligned} \textbf{t}=\frac{d_1}{4} \hat{\textbf{a}}_z\times \Bigg [ (\alpha _{\mathrm {me.1}}^{\textrm{co}}+\alpha _{\mathrm {me.2}}^{\textrm{co}}) \textbf{E}_{\textrm{t}}+(\alpha _{\mathrm {mm.1}}^{\textrm{co}}+\alpha _{\mathrm {mm.2}}^{\textrm{co}}) \textbf{H}_{\textrm{t}}\Bigg ] \end{aligned}$$

And for the toroidal moment *g*, we have,

21 $$\begin{aligned} \textbf{g}=\frac{d_2}{4} \hat{\textbf{a}}_z\times \Bigg [ (\alpha _{\mathrm {ee.1}}^{\textrm{co}}+\alpha _{\mathrm {ee.2}}^{\textrm{co}}) \textbf{E}_{\textrm{t}}+(\alpha _{\mathrm {em.1}}^{\textrm{co}}+\alpha _{\mathrm {em.2}}^{\textrm{co}}) \textbf{H}_{\textrm{t}}\Bigg ] \end{aligned}$$

These two Eqs. (20 and 21) can be simplified into the following equations:

22 $$\begin{aligned} \textbf{t}&=\frac{d_1}{4} \Bigg [(\alpha _{\textrm{me}}^{\mathrm {+co}}) (E_x \hat{\textbf{a}}_y - E_y \hat{\textbf{a}}_x)+(\alpha _{\textrm{mm}}^{\mathrm {+co}}) (H_x \hat{\textbf{a}}_y - H_y \hat{\textbf{a}}_x) \Bigg ] \end{aligned}$$

23 $$\begin{aligned} \textbf{g}&=\frac{d_2}{4} \Bigg [(\alpha _{\textrm{ee}}^{\mathrm {+co}}) (E_x \hat{\textbf{a}}_y - E_y \hat{\textbf{a}}_x)+(\alpha _{\textrm{em}}^{\mathrm {+co}}) (H_x \hat{\textbf{a}}_y - H_y \hat{\textbf{a}}_x) \Bigg ] \end{aligned}$$

The power generated due to the interaction of radiation fields and the chiral element pair is assumed to be determined by the Eq. (23),

24 $$\begin{aligned} P=-\frac{\omega }{2} \text {Im} \{(\textbf{p}+j\beta \textbf{t}) \cdot \textbf{E}_{\textrm{t}}^{*}+(\textbf{m}+j\beta \textbf{g}) \cdot \textbf{H}_{\textrm{t}}^{*}\} \end{aligned}$$

The first term in Eq. (23) is equal to,

25 $$\begin{aligned} \textbf{p} \cdot \textbf{E}_{\textrm{t}}^{*}=(\alpha _{\textrm{ee}}^{\mathrm {-co}})\left( E_{x}E_{x}^{*}+ E_{y}E_{y}^{*}\right) +(\alpha _{\textrm{em}}^{\mathrm {-co}})\left( E_{x}^{*}H_{x} +E_{y}^{*}H_{y}\right) \end{aligned}$$

The coefficient $\alpha _{\textrm{ee}}^{\mathrm {-co}}$ is used to express the relationship between the transverse electric field and the resultant electric dipole moments of the first element and the second element, which are represented by the difference $\alpha _{\textrm{ee}}^{\mathrm {-co}}$ and $\alpha _{\textrm{ee,1}}^{\textrm{co}}$ in Eq. (16). Similarly, $\alpha _{\textrm{ee,2}}^{\textrm{co}}$ is the coefficient for expressing the relationship between the transverse magnetic field and the resultant electric dipole moments of the first element and the second element, which are represented by the difference $\alpha _{\textrm{em,1}}^{\mathrm {-co}}$ and $\alpha _{\textrm{em,2}}^{\mathrm {-co}}$ in Eq. (16). The third expression, after performing inner product multiplication with the resultant magnetic dipole moments of two chiral elements and the mixed component of the transverse radiated magnetic field, is given by Eq. (25).

26 $$\begin{aligned} \textbf{m} \cdot \textbf{H}_{\textrm{t}}^{*}=(\alpha _{\textrm{me}}^{\mathrm {-co}}) \left( E_{x} H_{x}^{*}+ E_{y} H_{y}^{*} \right) +(\alpha _{\textrm{mm}}^{\mathrm {-co}})\left( H_{x}H_{x}^{*}+ H_{y}H_{y}^{*}\right) \end{aligned}$$

Calculating the second term in the general power relation (23) gives the result,

27 $$\begin{aligned} j\beta \textbf{t}\cdot \textbf{E}_{\textrm{t}}^{*} =j\beta \frac{d_1}{4}\bigg [\alpha _{\textrm{me}}^{\mathrm {+co}} (E_{x}E_{y}^{*} -E_{x}^{*}E_{y})+ \alpha _{\textrm{mm}}^{\mathrm {+co}} (E_{y}^{*}H_{x}-E_{x}^{*}H_{y})\bigg ] \end{aligned}$$

Calculating the fourth term in the general power relation (23) gives the result,

28 $$\begin{aligned} j\beta \textbf{g}\cdot \textbf{H}_{\textrm{t}}^{*}=j\beta \frac{d_2}{4} \bigg [ \alpha _{\textrm{ee}}^{\mathrm {+co}} (E_{x}H_{y}^{*}-E_{y}H_{x}^{*} )+\alpha _{\textrm{em}}^{\mathrm {+co}} (H_{x}H_{y}^{*} -H_{x}^{*}H_{y})\bigg ] \end{aligned}$$

Implementing circular polarization conditions for the radiant field in the form of and substituting the four computed terms in the power Eq. (23) yields the result,

29 $$\begin{aligned} P= & {} -\frac{\omega }{2} |\textbf{E}_{\textrm{t}}|^{2} \text {Im} \Bigg \{\alpha _{\textrm{ee}}^{\mathrm {-co}}-\frac{j}{2}\alpha _{\textrm{em}}^{\mathrm {-co}}\left( \frac{1}{Z_{TM}}+\frac{1}{Z_{TE}}\right) +\beta \frac{d_1}{4}\alpha _{\textrm{me}}^{\mathrm {+co}}+j\beta \frac{d_1}{4}\alpha _{\textrm{mm}}^{\mathrm {+co}} \frac{1}{2}\left( \frac{1}{Z_{TM}}+\frac{1}{Z_{TE}}\right) \nonumber \\{} & {} \qquad \qquad +\frac{j}{2}\alpha _{\textrm{me}}^{\mathrm {-co}} \left( \frac{1}{Z^{*}_{TM}}+\frac{1}{Z^{*}_{TE}}\right) +\frac{1}{2}\alpha _{\textrm{mm}}^{\mathrm {-co}}\left( \frac{1}{Z_{TM}^{2}}+\frac{1}{Z_{TE}^{2}}\right) +j\beta \frac{d_2}{4}\alpha _{\textrm{ee}}^{\mathrm {+co}}\left( \frac{1}{Z^{*}_{TM}}+\frac{1}{Z^{*}_{TE}}\right) \nonumber \\{} & {} \qquad \qquad -\beta \frac{d_2}{4}\alpha _{\textrm{em}}^{\mathrm {+co}}\frac{1}{2}\left( \frac{1}{Z_{TM}Z^{*}_{TE}}-\frac{1}{Z^{*}_{TM}Z_{TE}}\right) \Bigg \} \end{aligned}$$

We simplify the real part of some expressions and we have,

30 $$\begin{aligned} P= & {} -\frac{\omega }{2} |\textbf{E}_{\textrm{t}}|^{2} \Bigg [ \text {Im} \left\{ \alpha _{\textrm{ee}}^{\mathrm {-co}}\right\} -\alpha _{\textrm{em}}^{\mathrm {-co}} \frac{1}{2}\text {Re}\left\{ \frac{1}{Z_{TM}}+\frac{1}{Z_{TE}} \right\} +\alpha _{\textrm{me}}^{\mathrm {+co}}\frac{d_1}{4}\text {Im} \{\beta \}+j\alpha _{\textrm{mm}}^{\mathrm {+co}}\frac{d_1}{8}\text {Im}\left\{ \beta \left( \frac{1}{Z_{TM}}+\frac{1}{Z_{TE}}\right) \right\} \nonumber \\{} & {} \qquad \qquad +\alpha _{\textrm{me}}^{\mathrm {-co}} \frac{1}{2}\text {Re}\left\{ \frac{1}{Z^{*}_{TM}}+\frac{1}{Z^{*}_{TE}} \right\} +\text {Im}\{\alpha _{\textrm{mm}}^{\mathrm {-co}}\}\frac{1}{2}\text {Re}\left\{ \frac{1}{Z_{TM}^{2}}+\frac{1}{Z_{TE}^{2}}\right\} +j\alpha _{\textrm{ee}}^{\mathrm {+co}}\frac{d_2}{8}\text {Im}\left\{ \beta \left( \frac{1}{Z^{*}_{TM}}+\frac{1}{Z^{*}_{TE}}\right) \right\} \nonumber \\{} & {} \qquad \qquad +\alpha _{\textrm{em}}^{\mathrm {+co}}\frac{d_2}{8} \text {Im}\left\{ \beta \left( \frac{1}{Z^{*}_{TM}Z_{TE}}-\frac{1}{Z_{TM}Z^{*}_{TE}}\right) \right\} \Bigg ] \end{aligned}$$

The obtained general form in Eq. (23) can be examined for various conditions of radiant waves, including propagating or attenuating waves. If we assume that the conditions for $\beta$ are according to Eq. (30) and the impedance conditions are given by Eq. (31),

31 $$\begin{aligned} \beta= & {} nk_0=(n^\prime +jn^{\prime \prime })k_0 \end{aligned}$$

32 $$\begin{aligned} Z_{\textrm{TM}}= & {} \frac{\beta }{\omega \epsilon _{0}}=\eta _0 n \nonumber \\ Z_{\textrm{TE}}= & {} \frac{\omega \mu _{0}}{\beta }=\frac{\eta _0}{n} \end{aligned}$$

The special case where $n=1+j$ will simplify Eq. (29) to the form (Eq. 32) in the end.

33 $$\begin{aligned} P= & {} -\frac{\omega }{2} |\textbf{E}_{\textrm{t}}|^{2} \Bigg [ \text {Im} \left\{ \alpha _{\textrm{ee}}^{\mathrm {-co}}\right\} -\alpha _{\textrm{em}}^{\mathrm {-co}}\left( \frac{3}{4\eta _0}\right) \nonumber +\alpha _{\textrm{me}}^{\mathrm {+co}}\left( \frac{d_1k_0}{4}\right) \nonumber \\{} & {} +\alpha _{\textrm{mm}}^{\mathrm {+co}}\left( \frac{d_1k_0}{4\eta _0}\right) +\alpha _{\textrm{me}}^{\mathrm {-co}} \left( \frac{3}{4\eta _0}\right) +\alpha _{\textrm{ee}}^{\mathrm {+co}}\left( \frac{d_2k_0}{4\eta _0}\right) +\alpha _{\textrm{em}}^{\mathrm {+co}}\left( \frac{d_2k_0}{4\eta _0^2}\right) \Bigg ] \end{aligned}$$

## Results

### Absorber surface simulation

In this section, the results obtained from the simulation of a single cell of absorber surfaces are presented. The structure considered for the simulation is introduced and theoretically analyzed in pervious section. These simulations were conducted using the CST Studio Suite software. The purpose of these simulations is to achieve an optimal design and incorporate the findings into the theoretical analysis discussed in pervious chapter.

**Figure 9:**
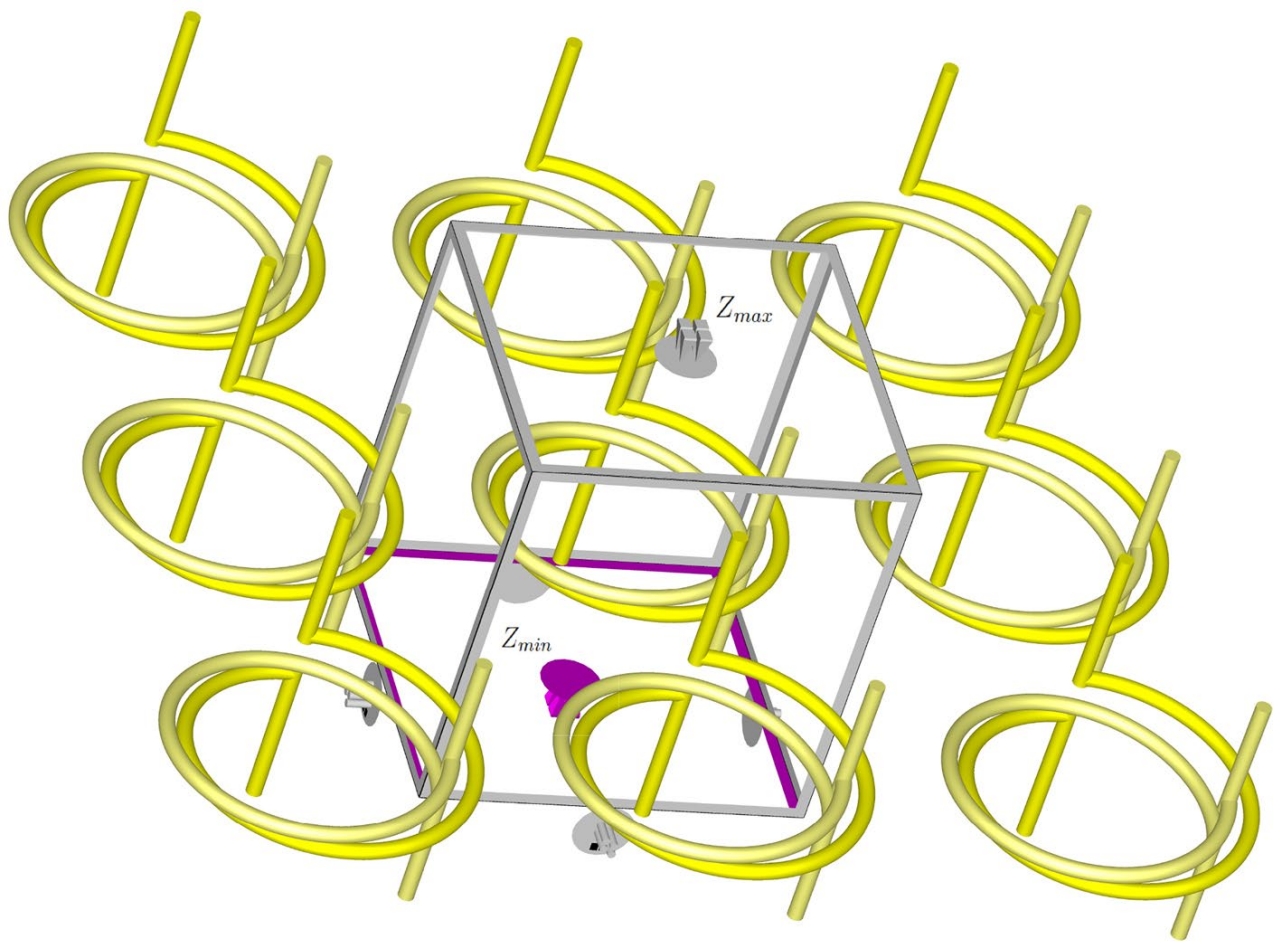
The simulation conditions of the simple desired structure in the CST Studio Suite software.

For the simulation, the proposed structure for a single cell is enclosed within a waveguide. The lateral walls of the waveguide are assigned periodic boundary conditions, while the top and bottom walls are fed with Floquet ports labeled as $Z_{max}$ and $Z_{min}$ as shown in Fig. 9. It is implicit that the incident sheet wave is assumed to have circular polarization based on the assumption discussed in pervious section. The distance of the structure from the lateral walls in each simulation is chosen such that the lattice constant *a*, which is the distance between the centers of each unit cell, is maintained. The Floquet ports are exclusively used in the simulation of planar periodic structures.

According to the Eq. (6), we randomly select an interval for the length. Please note that we have not yet calculated the optimal length as we require the values of *t* and $\delta$ (Fig. 8). Here, for a number of lengths, we find these values for a more optimized state. It is important to note that these results will not currently provide the best energy absorption percentage.

**Figure 10:**
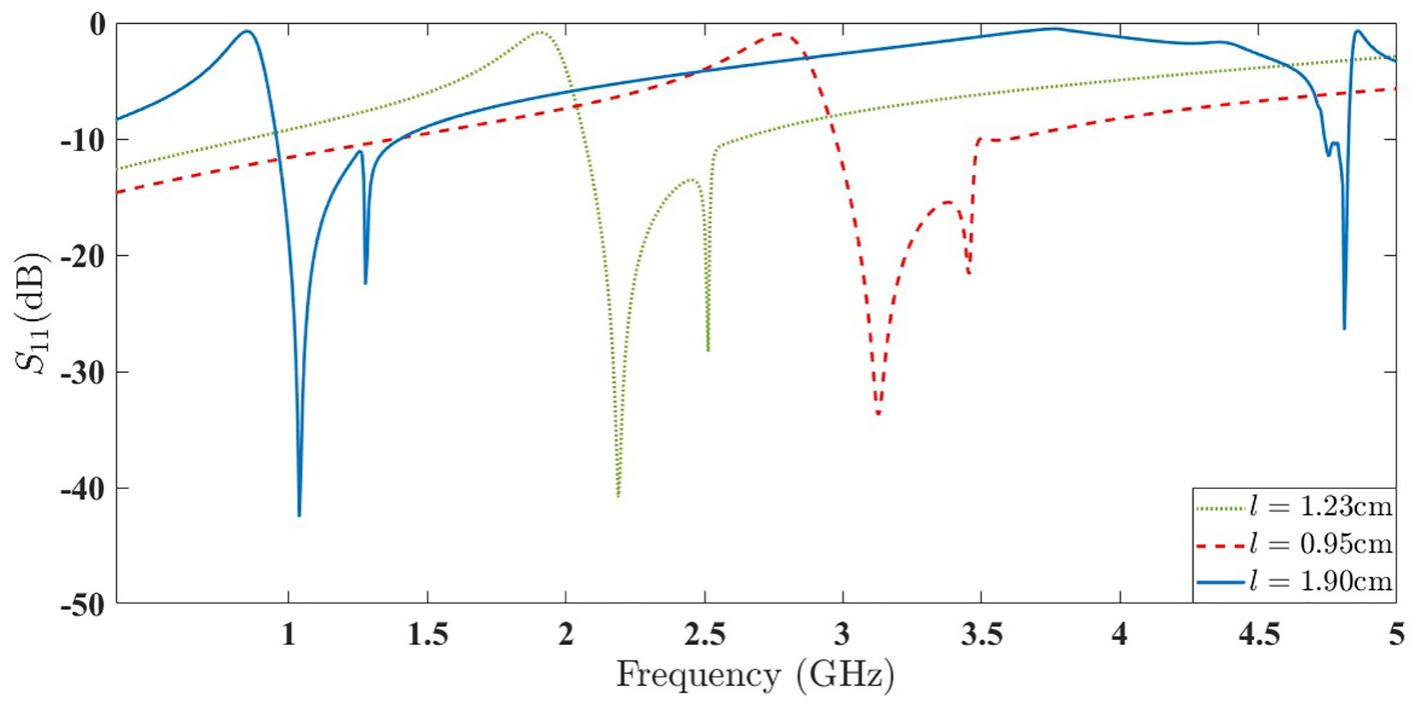
The scattering parameters of the three simulated structures.

A simulated sample of an optimized structure with $l=1.9$ cm, according to Eq. (6) from the Ref.^31^, exhibits the best performance in energy harvesting at a frequency of 1 GHz. Additionally, two smaller samples with $l=1.23$ cm and $l=0.95$ cm, with an amplification factor of 1.03 for the second element compared to the first element and a rotation angle of 25 degrees for the second element relative to the first element, as shown in Fig. 10, will yield results. The scatter parameter plots for these structures, shown in Fig. 10, exhibit noticeable movements similar to the results observed in structures with stimulated Tröger’s motifs.

**Figure 11:**
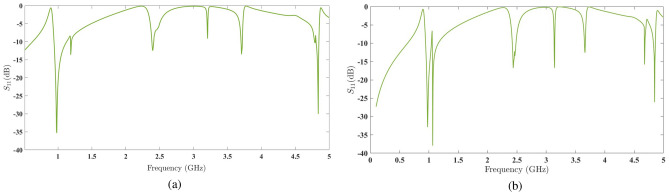
(**a**) The scattering parameters of the simulated structure with a 2% amplification factor. (**b**) The scattering parameters of the simulated structure with a 5% amplification factor.

Furthermore, the sample with a length of $l=1.9$ cm exhibits significant behavior in another region. We then conduct a case study on this structure to reach a general conclusion about the impact of separating the second element from the first element on the generation of specific resonances with high quality factors. Attention to the scattering parameter plots in Fig. 11, simulated respectively for amplification factors of $t=1.02$ and $t=1.05$ with a fixed rotation angle of $\delta =25^\circ$, reveals that the effect of the amplification factor t on the frequency region around 1 GHz is much more significant than its effect on the region around 4.8 GHz. The latter region is highly dependent on variations in the rotation angle $\delta$.

### The first resonant frequency with different amplification factors

The first frequency region, as discussed earlier, is highly dependent on the amplification factor *t* and relatively independent of the rotation angle $\delta$. By varying the amplification factor, one can achieve an optimal result for energy harvesting efficiency in this frequency region.

### The second resonant frequency with a unit amplification factor

To systematically study the impact of the amplification factor of the second element,*t* , and the rotation angle between the two chiral elements,$\delta$ , the results obtained from the structure shown in Fig. 8 with a unit amplification factor, $t=1$, for various rotation angles $\delta$ will provide valuable insights in this regard.

**Figure 12:**
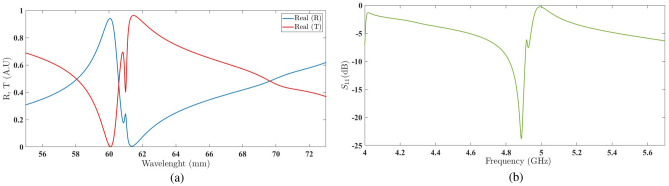
(**a**) The real values of the reflection coefficient and transmission coefficient of the structure embedded with a unit amplification factor and a rotation angle of 6^∘^. (**b**) The scattering parameter in the second frequency region with a unit amplification factor and a rotation angle of 6^∘^.

The graph in Fig. 12a represents the real values of the reflection coefficient and transmission coefficient of the structure embedded with a unit amplification factor and a rotation angle of six degrees. The observed discontinuities in the second frequency region, from 60 to $62\,\hbox {mm}$ wavelength, indicate the presence of a resonance in the reflection coefficient, as shown in Fig. 12b, for the scattering parameter $Z_{max}$.

**Figure 13:**
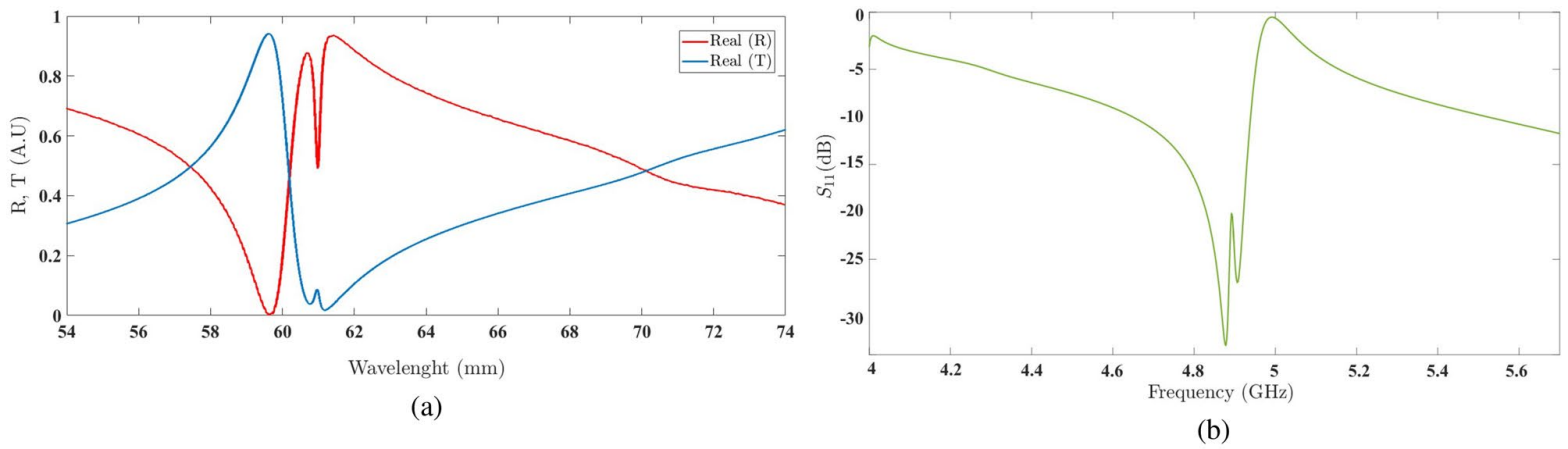
(**a**) The real values of the reflection coefficient and transmission coefficient of the structure embedded with a unit amplification factor and a rotation angle of 8^∘^ . (**b**) The scattering parameter in the second frequency region with a unit amplification factor and a rotation angle of 8^∘^.

Increasing the rotation angle to eight degrees results in a shift in the discontinuities of the real values of the reflection and transmission coefficients towards longer wavelengths, as shown in Fig. 13a. Consequently, the scattering plot is also shifted towards lower frequencies, including the resonant frequency of the second frequency region (at $4.8\,\hbox {GHz}$), as depicted in Fig. 13b.

**Figure 14:**
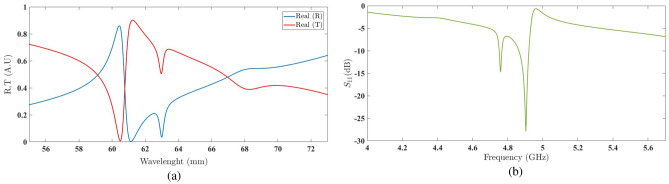
(**a**) The real values of the reflection coefficient and transmission coefficient of the structure embedded with a unit amplification factor and a rotation angle of 18^∘^. (**b**) The scattering parameter in the second frequency region with a unit amplification factor and a rotation angle of 18^∘^.

This behavior observed in the shift of discontinuities, as repeated in the case of $\delta =18^\circ$ (as shown in Fig. 14, provides us with a suitable insight into the impact of varying the rotation angle of the second chiral element. With such an understanding, it is possible to adjust the discontinuities to achieve the most desirable resonance in the second frequency region.

**Figure 15:**
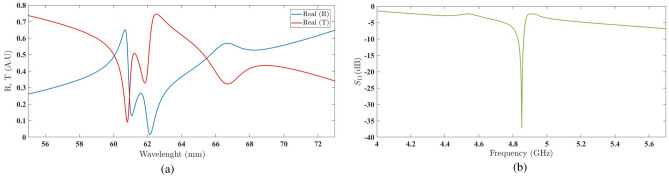
(**a**) The real values of the reflection coefficient and transmission coefficient of the structure embedded with a unit amplification factor and a rotation angle of $30^\circ$ . (**b**) The scattering parameter in the second frequency region with a unit amplification factor and a rotation angle of $30^\circ$.

The graph in Fig. 15a illustrates the successful attempt to achieve the desired resonance with a rotation angle of $\delta =30^\circ$. The scattering parameter plot in Fig. 15b confirms this accomplishment.

### Finding the maximum absorbed power

For numerical analysis, the normalized form of the obtained equation for the specified constrained conditions in Eq. (32) will be considered as $\widetilde{P}$ in Eq. (33).

34 $$\begin{aligned} \tilde{P}= & {} \omega \Bigg [ \text {Im} \left\{ \alpha _{\textrm{ee}}^{\mathrm {-co}}\right\} -\alpha _{\textrm{em}}^{\mathrm {-co}}\left( \frac{3}{4\eta _0}\right) +\alpha _{\textrm{me}}^{\mathrm {+co}}\left( \frac{d_1k_0}{4}\right) +\alpha _{\textrm{mm}}^{\mathrm {+co}}\left( \frac{d_1k_0}{4\eta _0}\right) +\alpha _{\textrm{me}}^{\mathrm {-co}} \left( \frac{3}{4\eta _0}\right) +\alpha _{\textrm{ee}}^{\mathrm {+co}}\left( \frac{d_2k_0}{4\eta _0}\right) \nonumber \\{} & {} \qquad +\alpha _{\textrm{em}}^{\mathrm {+co}}\left( \frac{d_2k_0}{4\eta _0^2}\right) \Bigg ] \end{aligned}$$

The variable $\tilde{P}$ can be modeled in **MATLAB** software for different values of *n*, where *n* relates the length of the first chiral element in the unit cell design to a specific wavelength $\lambda$ according to Eq. (34).

35 $$\begin{aligned} l_1=\frac{n}{1000}\lambda \end{aligned}$$

**Figure 16:**
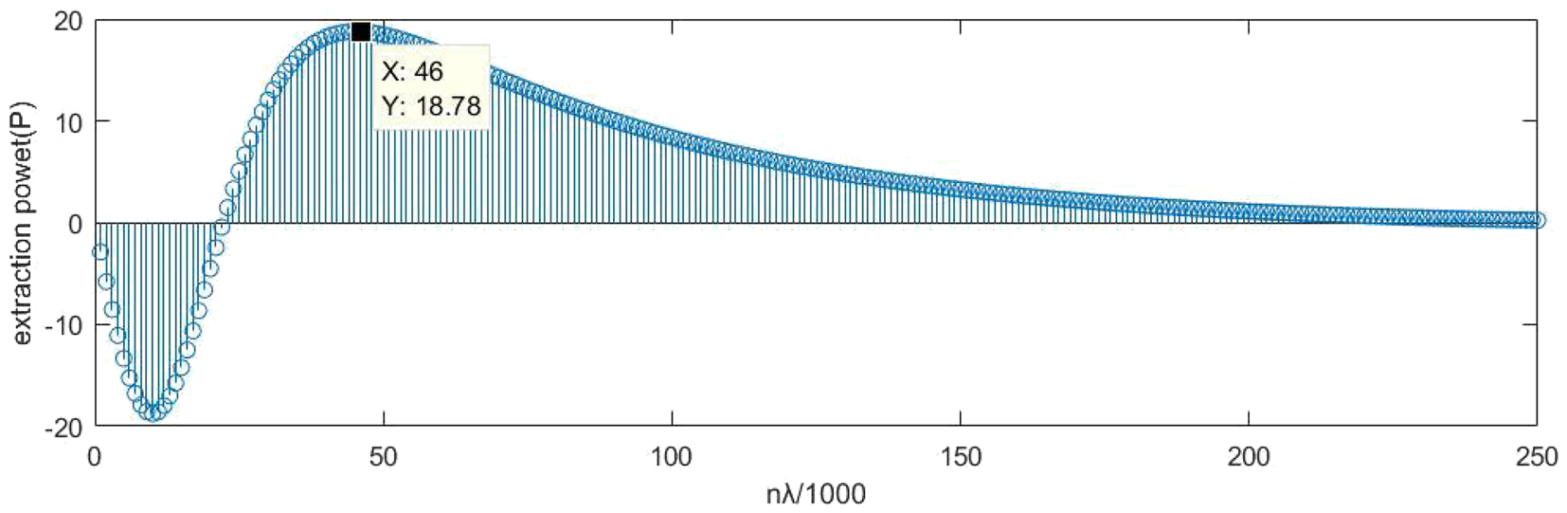
The obtained values for $\tilde{P}$ at each *n* in the range of 1 to 250.

The algorithm described in the Fig. 16 is executed in the **MATLAB** software to obtain the values of $\tilde{P}$ for different values of *n*, using a specific wavelength $\lambda =21.12$ cm. The resulting values of $\tilde{P}$ for each *n* in the range of 1 to 250, which covers up to a quarter of the wavelength, are plotted as a bar graph.

The obtained values indicate that the maximum value for the function $\tilde{P}$ occurs at $n=45$. Comparing this finding with the optimal chiral element length formula in Eq. (34) for the single chiral element at $\lambda =21.12$ cm suggests the achievement of an improved and generalized result for two elements within a unit cell. Furthermore, the use of a optimal length value of $l_1=1_{opt} = 0.97$cm for the first chiral element, where the magnification factor is $t=1.09$ and the rotation angle is $\delta =30^\circ$, confirms the optimal value for the desired resonance in the first frequency range, as determined by CST simulation.

Finally, considering the Ref.^35^ and the absorption power relation as follow,

36 $$\begin{aligned} PL = S^2 \eta _0 |E_0|^2 (1 - |R|^2 - |T|^2) \end{aligned}$$

**Figure 17:**
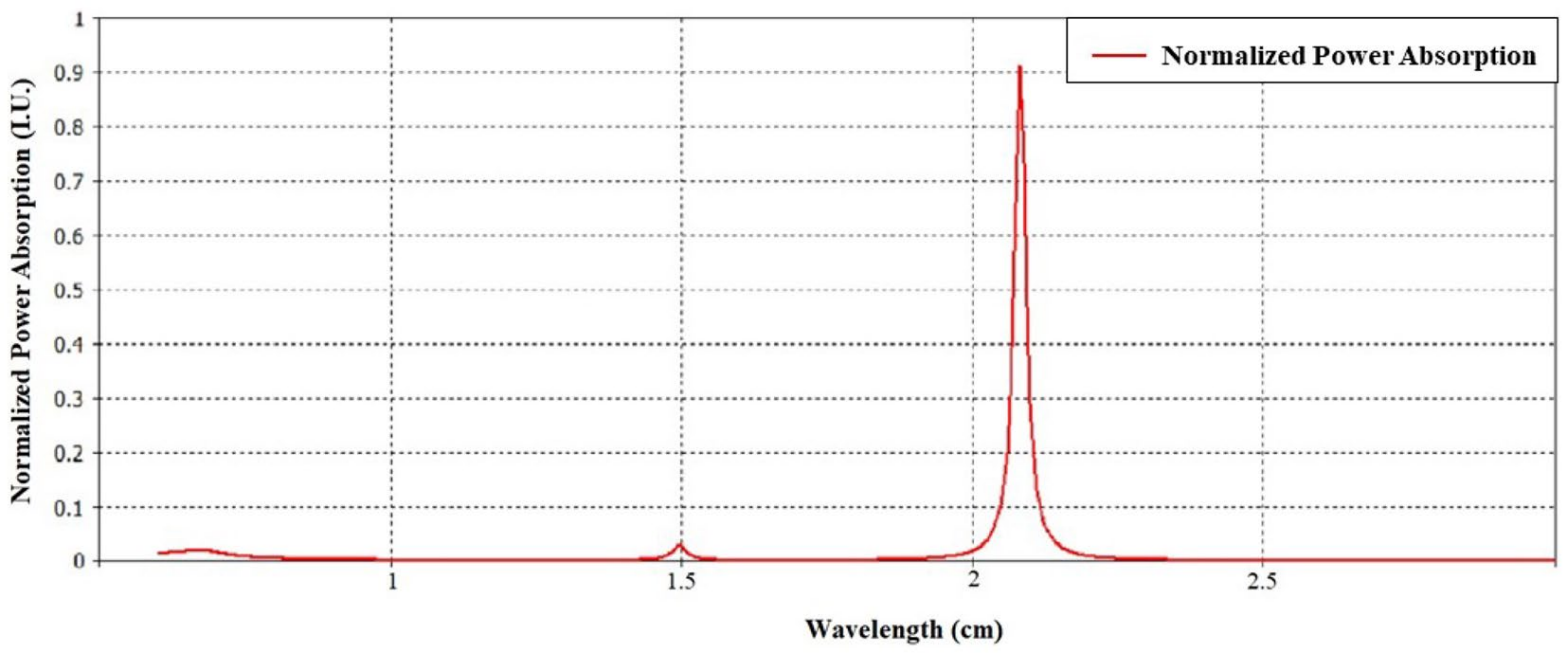
Normalized power absorption based on wavelength.

The normalized absorption power graph has been shown in Fig. 17.

As it’s obvious from Fig. 17, The normalized absorption power for $\lambda =2.112\,\hbox {cm}$ is over than 0.9 which has been improved relative to Ref.^35^ as a bianisotropic unit cell without toroidal configuration. The method’s versatility and flexibility suggest that it can be extended to various other structures, indicating a level of universality in its approach to characterizing bianisotropic particles based on reciprocity and magneto-electric couplings^49^.

As shown in Table 1, we present the absorption efficiency and frequency for different references. The absorptivity percentages and corresponding frequencies (in GHz) are listed alongside relevant references.

**Table 1:** Comparison of metasurface absorbers: absorption efficiency and frequency ranges.

Sl. no.	Year	Absorptivity (%)	Frequency (GHz)	Ref.
1	2015	$>80$	2.3	Ref.^50^
2	2016	$>90$	58.6	Ref.^51^
3	2016	$>80$	8.86	Ref.^52^
4	2016	$>90$	4	Ref.^53^
5	2015	$>90$	7.85	Ref.^54^
6	2016	$>90$	3.56	Ref.^55^
7	2016	$>90$	10.8	Ref.^56^
8	2017	$>90$	8.3	Ref.^57^
9	2020	$>90$	1.5	Ref.^30^
10	2023	$>90$	10.85	Ref.^58^
11	2024	$>96$	9.08	Ref.^59^
12	This work	$>92$	1.5	–

## Conclusion

In this study, we proposed and theoretically analyzed a novel design for a chiral metamaterial unit cell aimed at optimizing the absorption of electromagnetic waves. The unit cell consists of two chiral elements with a specific distance between them. The theoretical analysis involved expressing the polarizability coefficients and determining the interaction of radiated fields with the chiral elements. The focus was on achieving optimal energy harvesting by considering the toroidal moments generated by the electric and magnetic moments of the chiral elements.

The theoretical framework provided insights into the relationships between the design parameters, such as the amplification factor and rotation angle, and the generated toroidal moments. The derived equations allowed for the evaluation of the power generated due to the interaction of radiation fields with the chiral elements. The analysis considered circular polarization conditions and investigated the impact of varying parameters on the resonant frequencies.

The systematic study of resonant frequencies with different amplification factors and rotation angles provided valuable insights into the tunability of the proposed metamaterial structure. The observed shifts in resonances indicated the possibility of adjusting the design parameters to achieve specific resonant frequencies, contributing to the controllability of energy absorption.

The studied metasurface, with its narrow-band absorber properties, offers versatile application potential as a modulator across various fields^60^. Its ability to selectively filter and absorb specific frequencies benefits applications such as optical filters and spectroscopy^61^. Furthermore, the tunable absorption feature allows for real-time adjustment, making it suitable for sensors and optical devices requiring adaptive control^62^. In sensing and detection, its selective absorption enhances accuracy, while its high-fidelity modulation capability is crucial for precise light control in optical communication and imaging. Its compact and lightweight design further extends its utility, enabling integration into portable devices like wearables. Overall, these properties highlight its value as a modulator, providing spectral selectivity, tunability, sensitivity, high-fidelity modulation, and compactness for a wide range of applications.

## Data Availability

The datasets used and/or analysed during the current study available from the corresponding author on reasonable request.
